# Paving a Path Forward After an Unsuccessful GI Fellowship Match

**DOI:** 10.1007/s10620-025-09413-0

**Published:** 2025-09-30

**Authors:** Kevin Houston, Aimen Farooq, Mariam Naveed, Amy Oxentenko, Mohammad Bilal, Nikki Duong

**Affiliations:** 1 Virginia Commonwealth University Health System, Richmond, USA; 2 AdventHealth Orlando, Orlando, USA; 3 Mayo Clinic, Rochester, USA; 4 University of Colorado Health, Aurora, USA; 5 Stanford University, Stanford, USA

**Keywords:** Gastroenterology fellowship match, Unmatched, NRMP

## Abstract

The gastroenterology (GI) fellowship match is among the most competitive subspecialty matches in internal medicine, with approximately 35% of applicants going unmatched annually. For many trainees, not matching can feel like a significant personal and professional setback. However, this moment can also serve as an inflection point for growth and recalibration. Provided in this manuscript is a practical framework to support unmatched re-applicants in navigating the post-match period and strengthening their candidacy for future application cycles.

It is essential to acknowledge the emotional impact of an unsuccessful match and allow time for reflection before re-engaging in the process. While there may be a small number of open slots that do not fill and are open for a scramble-type process, this requires rapid action and access to all application materials to be readily sent, at a time when the unmatched applicant is still going through the disappointment of not matching. Re-applicants should seek mentorship and honest feedback to identify gaps in their applications and set clear goals for the upcoming year(s). Strategies to enhance re-applications include engaging in scholarly activity, pursuing structured research or clinical opportunities, strengthening letters of recommendation, and broadening the scope of program applications.

Ultimately, while not matching can be a difficult experience, many successful gastroenterologists have faced this challenge and gone on to excel. By reframing the setback as an opportunity to reflect, rethink, and restart, unmatched re-applicants can re-enter the fellowship match process with improved qualifications and a compelling story of perseverance.

## High-Level Points

- **Reflection is essential:** Take time to process the emotional impact of not matching and use this period for honest self-assessment and to establish short and long-term goals.
- **Setbacks are temporary and can foster growth:** Many successful gastroenterologists did not match on their first attempt; resilience, perseverance, and a proactive approach can change this setback into success.
- **Strategic growth matters:** Strengthen your candidacy through scholarly activity, structured clinical or research opportunities, and networking.

## Introduction

Gastroenterology is one of the most competitive specialties among Internal Medicine (IM) based upon the National Resident Matching Program (NRMP) statistics [1]. The gastroenterology (GI) and hepatology fellowship match is demanding and often brings reward, stress and uncertainty for applicants. While it is important for those participating in the match to have a back-up plan in case of an unsuccessful match, it may still come as a surprise. To some, not matching into a GI fellowship can feel like a major personal and professional setback.

Re-applicants should know they are not alone. Over the past decade, the number of fellowship applicants have increased at a rate that is exceeding the available positions in this country [1]. This manuscript provides practical steps for unmatched applicants to reflect, rethink and restart toward a successful fellowship match.

## Acknowledging the News and Initial Next Steps

### Reflect, Rethink, Restart

Before re-entering the “process,” it is important for the re-applicant to pause and acknowledge the emotional weight of not matching. Taking time to reflect is essential. It is important to resist the urge to internalize the experience, because feeling overwhelmed and upset is natural. It is important to keep a close support system of friends, family, colleagues, co-residents, and program leadership through the process. The match is unpredictable, and not matching does not lessen worth or accomplishments.

Experiencing a missed match carries many emotions: disappointment, self-doubt, frustration, even unexpected relief. These feelings are valid. This outcome does not minimize achievements thus far and should not define one’s future. A number of successful gastroenterologists did not match on their first attempt. As a community, we rarely speak openly about detours or setbacks, but these moments can be transformative. They teach resilience, courage and grit.

While there is no timeline on reflection, sooner or later the applicant will need to ask the question, “Do I want to continue to pursue a fellowship in GI?” Clarifying goals will shape how much time, energy, and effort to invest. An open and honest discussion with trusted friends and colleagues, professional advisors and mentors will be important to figure out the next steps which will typically be individualized to each re-applicant.

### Current Landscape for GI Fellowship Application

**Fig. 1:**
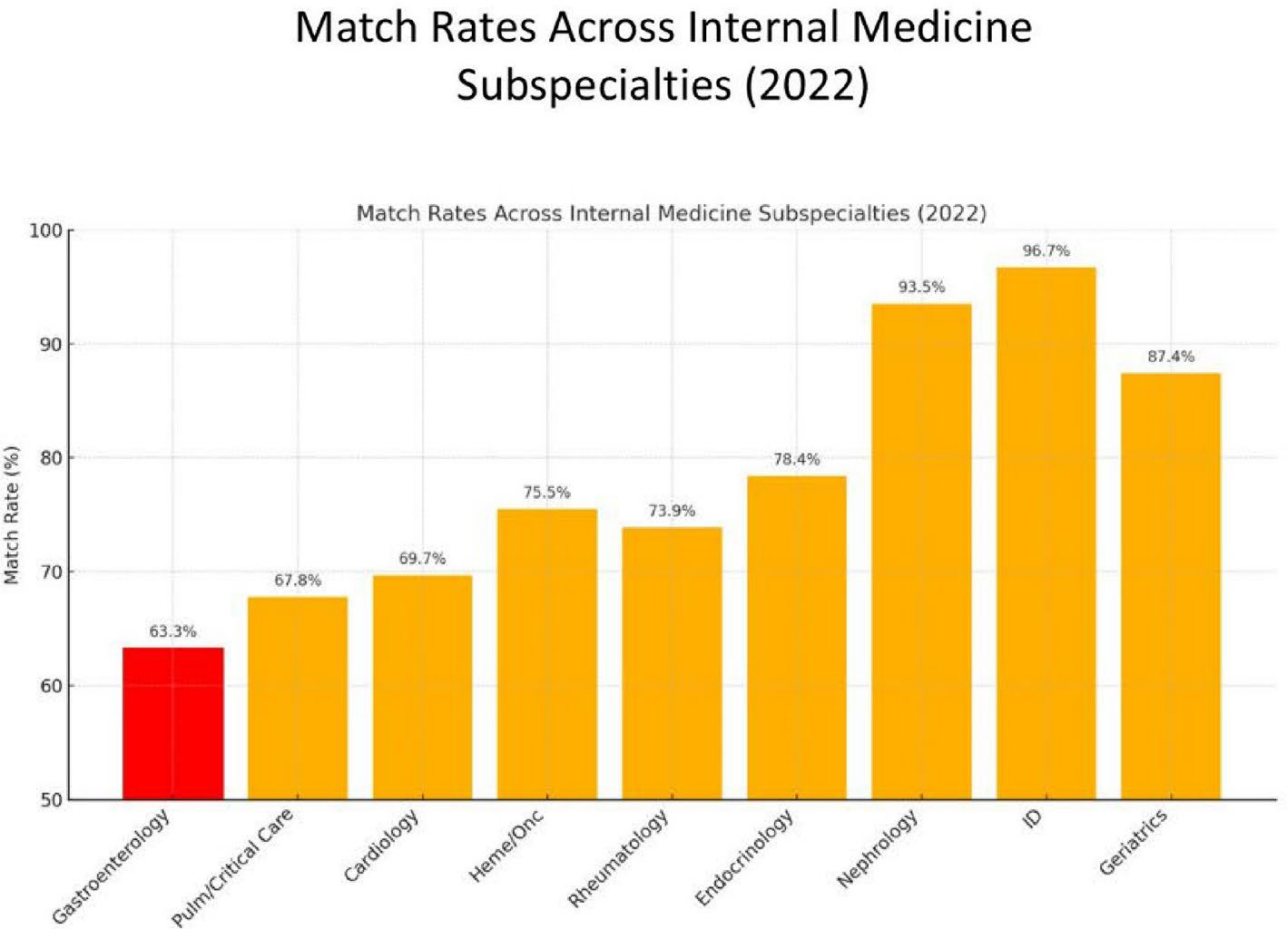
Internal medicine subspecialty match rates for the 2022 cycle

GI fellowship remains among the most competitive Internal Medicine subspecialties. Between 2010 and 2022, GI fellowship positions grew by approximately 4.6% per year, while the number of applicants increased by 3.8% per year [1]. As of 2022, GI had the highest specialty competitiveness ratio (SCR) among all IM subspecialties with 974 applicants and 616 GI positions—1.58 applicants per position (Fig. 1). This trend reflects both a strong interest in GI and perhaps a shortfall in available training positions.

For the last 5 years of the GI match, there has been an increase in programs and positions offered, yet the percent of unmatched applicants has remained relatively stable. In 2024, 688 of the 690 available positions were filled across 239 programs [2]. Of the 1064 applicants, 376 went unmatched (35% unmatched rate). From 2020 to 2024, GI unmatched rates remained steady at 35–37% (Table 1) [2].

**Table 1:**
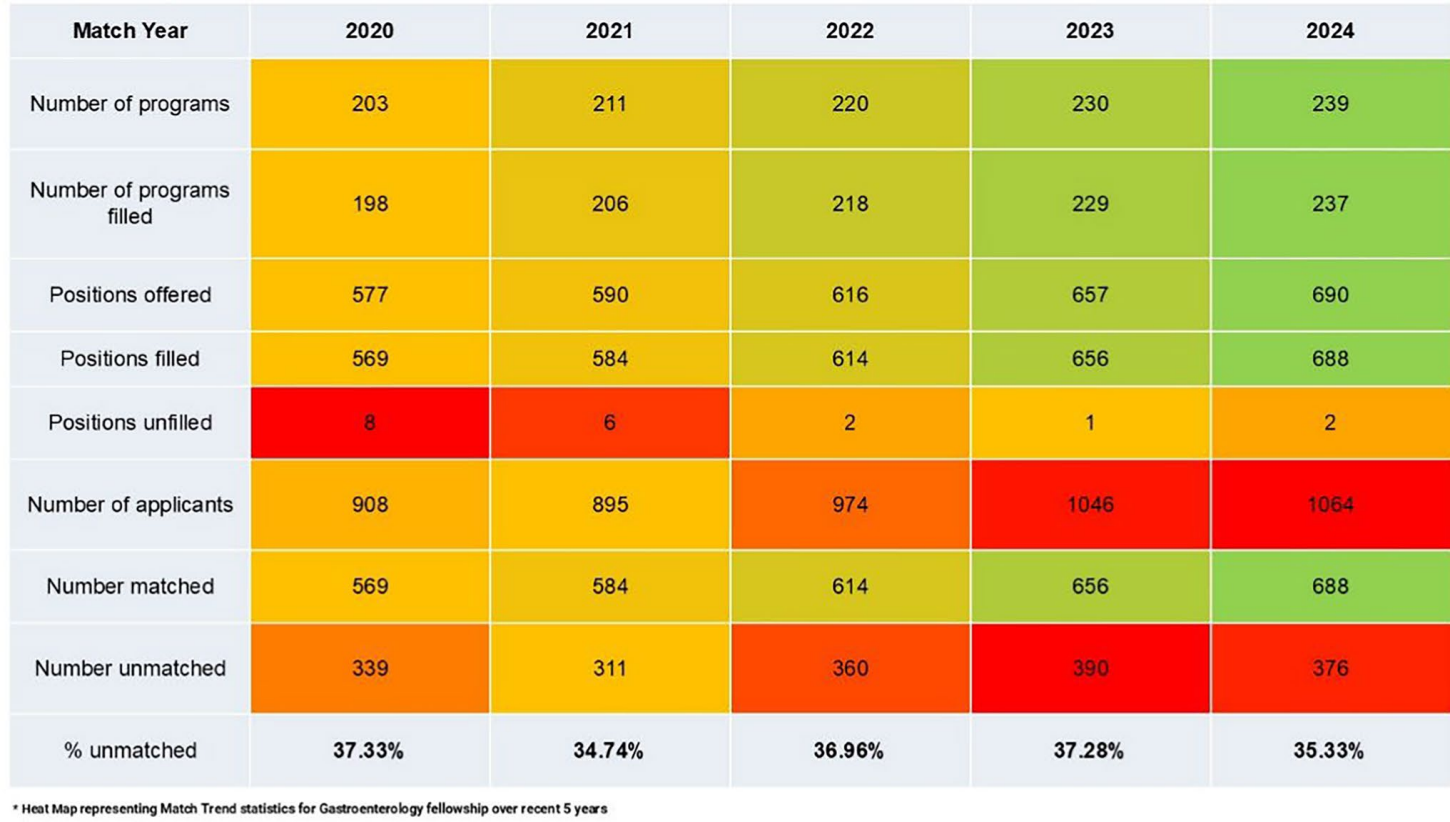
Gastroenterology match trends from 2020 to 2024

One way to appreciate the intensity of GI fellowship competitiveness is by comparing it to other subspecialties. For instance, from 2010 to 2021, cardiology fellowship applicants increased by 33%, positions by 46%, and the match rate stayed near 70% [3]. In 2021, there were 1.75 cardiology applicants for 1045 positions—roughly 1.51 applicants per position (similar to the SCR of 1.52 for GI in 2021). Hematology-oncology (HO) is another competitive field, though it has expanded training slots significantly. From 2009 to 2023, hematology-oncology positions grew 66% while applicants grew 34% [4], lowering the applicant-per-position ratio from 1.7 to 1.3.

The lesson from these comparisons is two-fold. First, match competitiveness varies widely, and many excellent applicants re-apply in many fields. Second, GI, while intensely competitive, is on par with or more selective than cardiology and HO currently. Recognizing these factors may help normalize the experience.

### General Timeline for the Unmatched Re-applicant

**Fig. 2:**
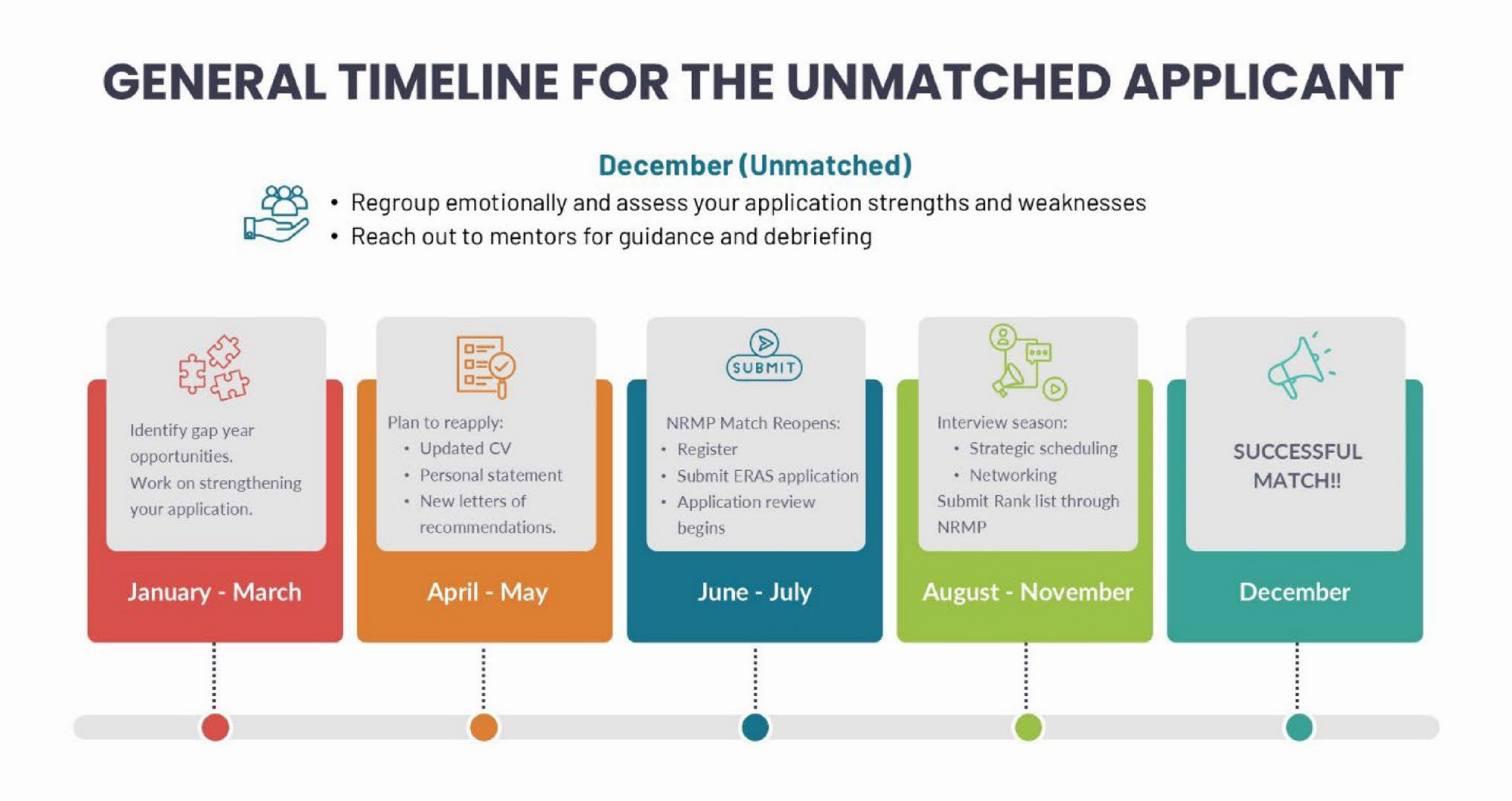
Suggested timeline for the unmatched GI fellowship applicant

Given that the match results are announced in December, if the re-applicant chooses to re-apply in July of the following year, they will have 7–8 months to work on improving their application as best as they can. Being aware of this short timeline and keeping to deadlines will ensure a smooth transition into the next cycle. Some key dates (exact dates will vary each year) are shown in Fig. 2, and these will be expanded upon in the discussion below.

### Application Review and Strategy Re-assessment

If possible, re-applicants should connect with their residency program director or the GI fellowship program directors (PDs) who interviewed them. They may provide constructive feedback about areas of improvement in their application. The key is to be open-minded and approach this with humility– the goal is not to assign blame, but to identify areas for growth. Talking with peers who matched and reviewing how they structured their applications can provide valuable insights and help refine strategies moving forward. The importance of peer mentorship cannot be underestimated. Reviewing one’s own ERAS entries, personal statement, and interview style can also highlight areas for improvement. Sometimes a relatively small fix (such as a stronger letter of recommendation) has the potential to make a significant difference. Also, ensuring there was alignment with strength and competitiveness as an applicant with the number of slots at the programs one applies to is crucial.

## Strategies for a Productive Gap Year

### Enhancing Scholarly Activity

**Fig. 3:**
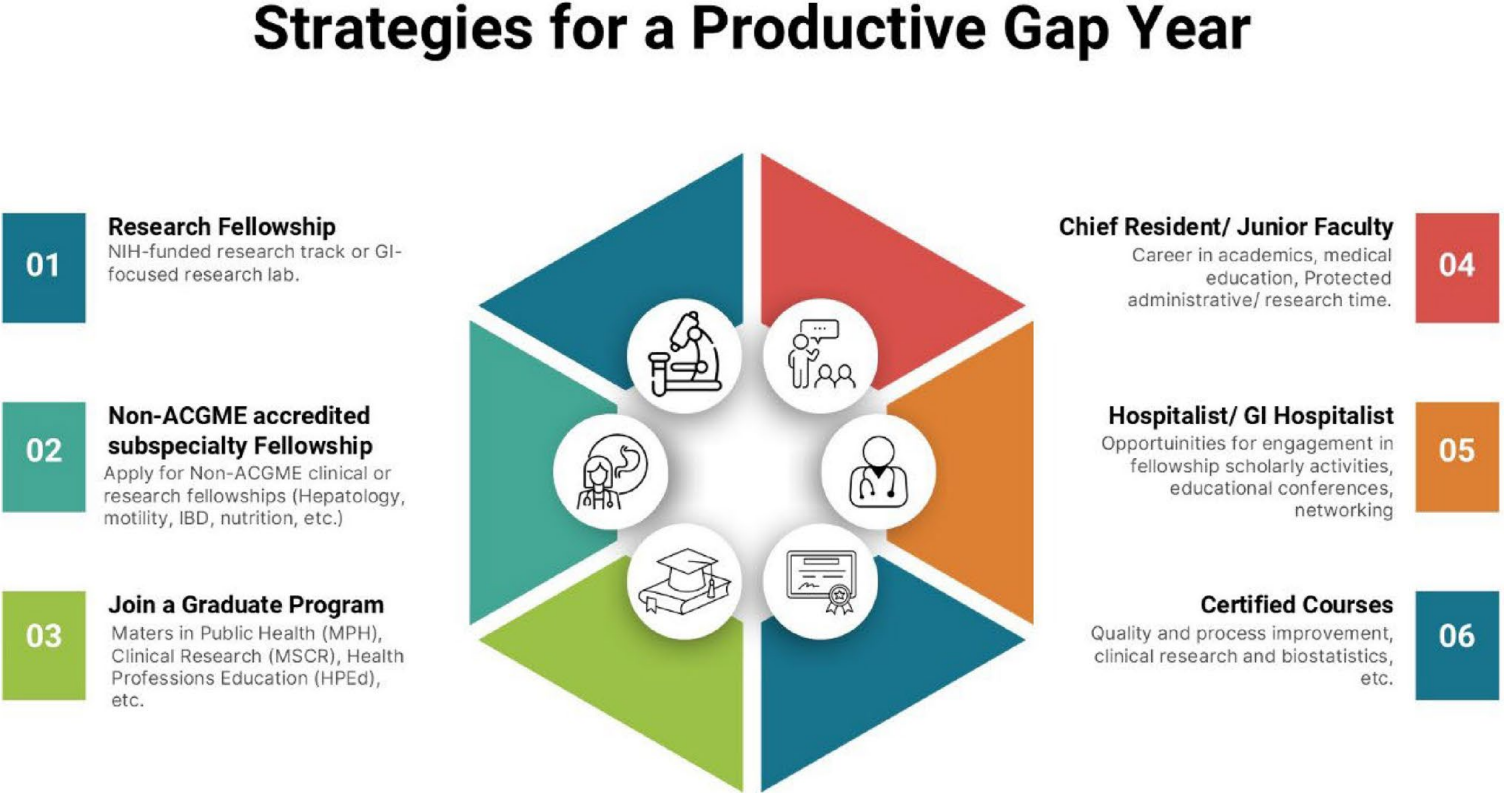
Gap year opportunities for the unmatched applicant

Scholarly activity is a key differentiator for many fellowship matches (Fig. 3). A national study of IM fellowship applicants found that publishing three or more peer-reviewed papers was strongly associated with matching (OR ~ 4.7 for matching if ≥ 3 papers) [5]. In other words, matched applicants had “significantly more” scholarly output (abstracts, presentations, publications) than those who did not. Re-applicants should consider dedicating the coming year to GI-related research: for example: joining a lab, converting past abstracts into publications, or initiating a clinical or quality improvement study.

A structured timeline and strong mentorship will be crucial, keeping in mind the timing surrounding the IRB approval process, data collection, and meeting submission dates.

### Enhancing Scholarly Activity: Consider a Research or Clinical Year

Many re-applicants use the “intervening year” strategically. Though this is not a comprehensive list, the three main pathway approaches are: hospitalist positions, research fellowships, or non-ACGME accredited subspecialty fellowships.

Some literature suggests that a protected research year is often the most successful strategy for re-applicants, as it provides time to engage in meaningful scholarly output [5]. In contrast, remaining in a purely clinical hospitalist role without an emphasis on scholarly activity may result in an unchanged application. Consider seeking opportunities such as NIH-funded research tracks or translational GI labs. In recent years, GI hospitalist or hospitalist-subspecialty scholar programs have emerged for internal-medicine board eligible/board certified physicians looking for direct GI clinical exposure. If a formal fellowship or chief year isn’t available, consider carving out a part-time research niche while working clinically. The key is to produce tangible outcomes (manuscripts, posters, grants) that can be shown on a CV and discussed during interviews.

Another option may be to seek out opportunities for junior faculty positions that allow negotiable protected time for research or administrative work while mentoring residents and medical students. This role can strengthen a profile in medical education and mentorship—qualities that are highly valued by fellowship programs and aligned with a long-term goal of an academic career. While many are post-GI fellowship training, some institutions may offer non-ACGME accredited subspecialty fellowship opportunities in hepatology, motility, inflammatory bowel disease (IBD), and nutrition. These entail engaging in a didactic curriculum, collaborating with GI fellows and faculty, further enhancing exposure to the field. It is important to inquire about outcomes of the previous fellows before selecting how to spend the upcoming year(s). This will offer invaluable insight into the quality and effectiveness of such a program and allow a path that best aligns with one’s needs and goals.

Do not neglect clinical standing! Re-applicants should continue performing strongly on wards and clinics during their time left in residency (if applicable)—as reputation matters. During this extra year, cultivate not only a stronger resume, but continue to be reliable, diligent, and compassionate in service. No amount of research output can compensate for being known as careless or difficult to work with.

If one chooses to take on additional responsibilities (i.e., chief resident role or an educational project), it must be done well–word travels quickly in academic medicine. If available and if it aligns with one’s interest, earning a GI-relevant certification (like courses in endoscopy or hepatology) or advanced degrees (Master’s in Clinical Research or Public Health, for instance) may also be helpful. These steps can show commitment to continuous learning and growth.

### Building a Network via Mentorship and Professional Engagement

Re-applicants do not have to navigate this process alone. Mentors and professional communities are key allies. An experienced mentor can provide advice, feedback, and sometimes even advocate for re-applicants. Many professional societies offer forums, travel grants, and opportunities for non-fellow trainees at national and regional meetings. It is the hope that with the growing interest in Gastroenterology programs expand both formal and informal mentorship opportunities for these trainees.

Re-applicants should aim to attend regional and national GI meetings (e.g., Digestive Disease Week, ACG’s Annual Meeting, AASLD’s The Liver Meeting). These meetings allow re-applicants to meet faculty and current fellows in person—an invaluable opportunity in the era of virtual interviews post COVID [6–8]. Introductions with GI program directors during meet-and-greets or poster sessions can demonstrate continued interest and can leave a memorable impression. In other words, every interaction is an opportunity to reinforce a strong commitment.

Volunteering and community involvement in GI-related work also strengthen an application.. Participating in patient advocacy events or GI-focused volunteer clinics not only enriches experiences but also provides networking opportunities. Local chapters of GI foundations (like the Crohn’s & Colitis Foundation or the American Liver Foundation) often hold events and seminars. Participating in these initiatives demonstrates a service-oriented mindset and expands one’s professional network.

## Practical Approaches When Re-applying

### Gather Strong Recommendations

Letters of recommendation (LORs) carry substantial weight in the fellowship application process, especially for re-applicants.

Asking mentors who know the applicant well and can speak to their improvements is a strong aspect of all re-applicants. A helpful approach is to meet with each potential writer: discuss goals and share an updated resume along with a new personal statement. Provide context such as what aspects to highlight (e.g., dedication to service, teamwork, research accomplishments, awards). If a re-applicant pursued a research year, the respective mentor’s letter should emphasize updated projects and a strong work ethic or perseverance. It is essential to have at least one LOR from a GI faculty member, as this signals GI-specific endorsement, and more GI-centric letters are even better across different experiences (e.g., clinical experiences, research, etc.). Conversely, if the applicant completed significant work with an individual but does not have a very strong letter from them, this could be a “red flag” and raise questions. A team of mentors and advisors can also advocate and reach out to their networks when interview season arrives. It is important to be strategic about who to ask for help, when to ask, and how to ask. Respect other’s time commitments, as asking for too many favors may be counterproductive.

### Expand Where to Re-apply

The rank list should be re-evaluated strategically. It may make sense to apply to more programs or to include smaller programs, those in different regions, or primarily research-focused tracks. Use NRMP and specialty society match data (like charting outcomes or program websites) to gage program fill rates and alumni career paths. If an applicant’s profile is stronger for research, they should target programs with NIH funding or hybrid schedules.

Be realistic about program fit. For instance, if most matched candidates at a program had more than 10 publications and were AMG (American Medical Graduates), and the re-applicant’s profile does not align with that, consider balancing the list with programs where recent fellows had comparable backgrounds. Discuss choices with mentors who understand the process. It would be important to reflect on whether you apply to entirely new programs versus a hybrid of some new versus some that were applied to in the first cycle—this may come down to trying to understand as best as possible what the issue, if any, may have been the first time.

### Telling a Story—Personal Statements and Interviews

For many enthusiastic applicants, the path to GI fellowship has been a long journey. Programs are searching for more than a resume with a long list of publications. They want individuals who are well-rounded and show strong potential to become thought leaders in the field.

The application is more than numbers and checklists—it’s a narrative. The re-applicant should use the personal statement to tell a cohesive and compelling story. Emphasize themes like growth, purpose, and perseverance. The best personal statements are authentic, forward-looking, and do NOT use artificial intelligence. If the applicant chooses to address the re-application, they should do so briefly and constructively. Avoid a tone of defeat; instead, frame things as learning experiences and resilience. Finally, have mentors or colleagues proofread it carefully—typos or questionable phrases can undermine an otherwise solid application.

Interview preparation is equally important. Be sure to practice your interview—how you present yourself and what you are going to say. Several resources exist for applicants [9, 10]. Be ready to openly discuss why one believes they went unmatched and what they have learned from this process. For example, “the last cycle didn’t go as planned, so I spent the year doing ‘X’, and I feel my application is stronger now.” Talk about specific projects or insights that one has gained. This will allow programs to assess maturity, insight and interpersonal skills. Applicants should continue to show enthusiasm, ask thoughtful questions about the program, and listen actively. Each interview is just as much an audition of character as it is of credentials.

## Conclusion

Not matching into GI fellowship is undeniably difficult, but it can serve as a pivotal moment for many re-applicants. Many successful gastroenterologists have walked this path and ultimately found even greater success by using the extra time wisely.

By understanding the competitiveness of the field, seeking mentorship, engaging with the GI community, and strategically refining one’s application, re-applicants can re-enter the match as a stronger, more polished candidate. Re-applicants should commit to their goals and let their passion for GI guide them forward. This detour may have been unexpected, but a temporary setback can ultimately mark the beginning of a long and successful career.

## Data Availability

No datasets were generated or analysed during the current study.
